# Assessment of primary health care workers’ knowledge and skill for COPD and asthma screening and diagnosis in Jos LGA of Plateau State

**DOI:** 10.11604/pamj.2022.41.21.29932

**Published:** 2022-01-10

**Authors:** Chundung Asabe Miner, Esther Awazzi Envuladu, Tolulope Olumide Afolaranmi, Basil Nwaneri Okeahialam, Ayuba Ibrahim Zoakah

**Affiliations:** 1 Department of Internal Medicine, Faculty of Clinical Sciences, College of Health Sciences, University of Jos, Plateau State, Nigeria

**Keywords:** COPD, asthma, primary healthcare workers, knowledge, screening, diagnosis, Nigeria

## Abstract

**Introduction:**

chronic obstructive pulmonary disease and asthma are the commonest of the group of Chronic Respiratory Diseases. Primary Health Care workers play a role in the prevention of these diseases. The aim of this study was to determine the knowledge and diagnostic skills of PHC workers on COPD and asthma in a local government area of Plateau State, Nigeria.

**Methods:**

it was a cross-sectional study conducted among 146 primary healthcare workers selected through a multistage sampling technique. Data was collected through a self –administered questionnaire. Data processing and analysis were done with Epi-Info epidemiological software. The categories of knowledge that were of interest were scored and graded. A confidence level of 95% was used and a p-value of <0.05 was considered significant for this study.

**Results:**

the mean age of respondents was 41.4 ± 10.1 years, junior community health extension workers made up the largest group and the mean years of service was 14.9 ± 8.9 years. The overall knowledge of COPD and asthma was fair in 68.5 % of health workers though the mean knowledge scores of asthma were statistically significantly higher than that of COPD (p = 0.000). Knowledge was found to be statistically significantly associated with age, sex and cadre. None of the respondents was able to operate a peak flow meter.

**Conclusion:**

the study concluded that though there was fair level of knowledge among the respondents on COPD and asthma, they lacked the necessary skills to screen for and diagnose these diseases.

## Introduction

Chronic respiratory diseases (CRDs) are a group of chronic diseases affecting the airways and other structures of the lungs [1]. They are ranked among the four commonest non-communicable diseases (NCDs) worldwide [2]. They were recognized in 2012 as a cause of significant disability and were responsible for 6.4% of all NCD deaths. The commonest of these CRDs worldwide are chronic obstructed pulmonary disease (COPD), asthma and occupational lung diseases. A survey across nine countries showed that respiratory symptoms are among the commonest causes of consultations in primary health care facilities with a range of 8.4%-37% [1].

Chronic obstructive pulmonary disease (COPD) is a group of progressive lung diseases that obstruct airflow. The symptoms develop slowly and over time, making it hard to perform routine tasks [3]. Asthma is a chronic disease characterized by recurrent attacks of breathlessness and wheezing, which vary in severity and frequency from person to person affecting both children and adults [4]. Though COPD and asthma have seemingly similar presentations, distinct differences exist between the two such as the age of presentation and reversibility in airway limitation [5]. These differences make it possible to screen and diagnose the two diseases at an early stage.

An estimated 210 million people are suffering from COPD and 300 million people from asthma [1]. There were 3 million deaths from COPD and 250,000 from asthma in 2005. In 2002, COPD was the fifth leading cause of death and is currently the fourth leading cause of death globally and is predicted to become the third by 2025 [6,7]. The deaths from both diseases are mainly in low- and middle-income countries (LMICs). Two nationwide surveys have been conducted on NCDs in Nigeria but both have covered only the south west region of the country and both surveys excluded CRDs [8,9]. However several studies in the country have shown a worrying presence of COPD and asthma in different populations in the country [10-13].

As the risk factors for these diseases are mostly preventable being mainly behavioural and modifiable, focusing on reducing the risk factors is one of the most important ways of controlling the diseases [2]. Global assessment made identified the main risk factors for COPD to be smoking and the ambient particulate matter followed by household air pollution, occupational particulates, ozone and second-hand smoke. Risk factors identified for asthma were smoking and occupational asthmagens [14]. Tools such as health education, screening, early diagnosis and treatment are all essential in reducing the morbidity and mortality that is associated with the occurrence of these diseases. Early detection of these diseases is economically advantageous as the cost of treatment and rehabilitation far outweighs that of prevention. These preventive strategies are implementable at the level of primary health care which is mostly manned by community health workers.

Community health workers are lower cadre staff that are predominantly found in the Primary Health Care (PHC) units of Nigeria. They are the frontline health managers who are expected to meet the needs of the larger population of Nigeria. Their role naturally includes health promotion and prevention and treatment of common ailments [15]. Their engagement so far has mainly been in the area of infectious diseases and maternal, newborn and child health [16]. However, they are still expected to be able to identify risk factors and screen for NCDs and be able to refer patients as indicated. COPD and asthma have been stated to be “under-recognised, under-diagnosed, undertreated and insufficiently prevented” [6]. For control measures to be effective, especially at the community level, there is a great need for this cadre of health workers to have adequate knowledge and skills in identifying members of the community who are either at risk or have developed COPD or asthma.

The risk factors for COPD and asthma have been documented in studies conducted within Nigeria and the study area [17-21]. Forming fertile ground among the citizens of Jos North LGA for the development of these diseases. It is pertinent for any current efforts in the control of NCDs to know if the frontline workers in our communities have the requisite knowledge and skill required to ensure that CRDs can be tackled at primary level of health care.

The use of a questionnaire with manual/sensor-based peak flow meter or COPD-6 vitalograph and spirometry has been adjudged the most reliable method for the screening and subsequent diagnosis of COPD and asthma [22-24]. A questionnaire with a minimum of 4 questions to detect symptoms and risk factors and a peak flow meter have been shown to be highly sensitive in detecting those with COPD and/or asthma [25]. A Peak Expiratory Flow Rate (PEFR) of < 70% is usually indicative. In the World Health Organization Package of Essential NCD Interventions (WHO PEN) minimum package guidelines designed for primary care in low resource settings, it recognizes that asthma and COPD can present with similar symptoms, in particular cough, difficult breathing, tight chest and/or wheezing [26]. A set of questions are designed that obtain information such as age, a previous diagnosis of the condition, age of onset of symptoms, history of allergies, smoking, inhalation of biomass smoke, sputum production and breathlessness. The health worker is then expected to take the PEF rate, administer salbutamol and repeat the measurement in 15 minutes. An improvement of PEF by 20% is asthma, less than that is COPD [26]. Guidelines for management are outlined with room for referral when there is no improvement or symptoms increased in severity.

The objective of the study was therefore to assess the level of knowledge and skill of PHC workers to identify risk factors, to screen and diagnose COPD and asthma.

## Methods

**Study design:** a cross-sectional design was used for the study.

**Study setting:** the study was conducted in Jos North Local Government Area (LGA) of Plateau State, Nigeria. The LGA is made up of 20 wards, 19 of which contain at least one of the 34 PHC centres located across the LGA. These facilities have a total of 532 health workers 210 of whom were identified to perform clinical functions [27].

**Study population:** the study population was made up of 146 primary health care workers selected from 24 primary health care facilities.

**Determination of study size:** the minimum sample size was calculated using Cochran´s formula [28].


$$n=\frac{z^{2}pq}{d^{2}}$$

The prevalence used for the calculation was obtained from a study that assessed the knowledge of health workers on asthma (53 % having good knowledge) [29]. The sample size was further modified using the formula for smaller populations [28].


$$n_{f}=\frac{n}{1}+\frac{n-1}{N}$$

The initial calculated minimum sample size (n) needed to be revised downwards because the estimated number of primary health workers of interest (N) from eligible facilities in the study area was 203. An additional 10% was added for non-response giving a minimum sample size of 146.

**Eligibility criteria:** these were health workers that were actively involved in clinical care and had been working for at least 6 months prior to the commencement of the study. Those whose duties were primarily administrative and those on leave (sick, annual or study) were excluded.

**Selection of study participants:** a multi-staged sampling technique was used to select participants. The first stage was the selection of Jos North LGA from the 7 LGAs that make up the Plateau North senatorial district through simple random sampling by balloting. The second stage was the inclusion of all the 19 wards out of the 20 wards of the LGA that had functional PHC centres. This was achieved using a list obtained from the Jos North Primary Health Care Department. For the third stage, an initial selection of one PHC facility per ward was done to ensure that all the 19 wards were covered. Where there was only one facility, it was automatically selected. Where there was more than one, a simple random sampling technique by balloting was used to select one facility. After selecting the initial 19 PHCs, an additional 5 facilities were selected from those that had not been initially selected through a simple random sampling technique by balloting in order to achieve the minimum sample size. For the final stage, all eligible respondents in each health facility were identified and selected through the facility duty roster and the assistance of the officer in charge to participate in the study. Ethical approval and participant consent: Ethical approval was obtained from the Jos University Teaching Hospital (JUTH) Human Research and Ethics Committee. Permission was obtained from the Plateau State Ministry of Health, the Local Government Chairman of Jos North LGA and the Director of PHC in Jos North. Written informed consent was obtained from each participant before inclusion in the study.

**Data collection method:** data was collected in July of 2018 over a period of 2 weeks. Data was collected with the use of a semi-structured self-administered questionnaire which included sections on respondent´s socio-demographic data, knowledge of asthma and COPD risk factors, screening methods and diagnosis and health worker skill evaluation. The Cronbach´s Alpha score for assessment of the tool´s internal validity was determined to be .84 and it was pre-tested among a similar population. Six research assistants were trained on the administration of the checklist and use of the peak flow meter to ensure uniformity on observations made. Peak flow meters were also made available for use to the research team to have at hand for use if required. Once eligible participants were identified and consent was obtained, a questionnaire was provided and they were given time and privacy to fill the questionnaire. Participants were required to demonstrate ability to use the peak flow meters once they indicated in the questionnaire that they knew how.

**Variables of interest:** independent variables were the socio-demographic characteristics, namely age, sex, cadre, the highest level of education, duration of practice, marital status, tribe, religion and previous training in screening for COPD and asthma. The outcome variables were knowledge of asthma and COPD, factors affecting knowledge and skill evaluation.

**Scoring and grading of knowledge:** knowledge was assessed with the scoring system used by Memon *et al*. [30]. For each question, correct answers were scored 1 mark while wrong answers or selecting “don´t know” were scored a 0 mark. There was a total of 28 marks for each of the knowledge sections on asthma and COPD, giving a total of 56 marks. The scores were then used for grading into “good, fair, poor” based on the following criteria: For either asthma or COPD; good was 20-28 marks, the fair was 14-19 marks and poor was 0-13 marks. For the combined total scores; good was 40-56 marks, fair was 28-39 marks and poor was 0-27 marks.

**Data analysis:** data were entered, cleaned and analysed using Epi-Info version 7.2.2.6. There were no missing data as questionnaires were cross-checked after being filled and participant phone numbers were stated on the questionnaires. The socio demographic profile of the health workers is presented in frequencies and percentages. Knowledge scores were graded and mean scores obtained. Chi-square test was used to determine the relationship between socio-demographics and knowledge of COPD and asthma. T-test was used for comparison of means. A confidence level of 95% was used for the study and a p-value of = 0.05 was considered statistically significant.

## Results

**Sociodemographic characteristics of participants:** the response rate for the study was 100%. The mean age of the health workers was 41.4 ± 10.1 years. A majority of the health workers were females. The cadre of junior community health extension workers (JCHEW) formed the largest group (35.6%) among the various cadres. The mean years of service was 14.9 ± 8.9 years. Most were married and their tribes were predominantly those that are indigenous to Plateau State. The majority (78.1%) were Christians and only 20 (13.7%) had been trained on CRDs and all of them stated that their training was in school (Table 1).

**Table 1:** socio-demographic characteristics of respondents

Variable	Frequency (N = 146)	Percentage (%)
**Age Group (years)**		
20 - 30	27	18.5
31 - 40	41	28.1
41 - 50	51	34.9
51 - 60	27	18.5
**Sex**		
Female	117	80.1
Male	29	19.9
**Cadre**		
Nurse/Midwife	34	23.3
CHO	10	6.9
CHEW	50	34.3
JCHEW	52	35.6
**Highest Level of Education**		
Certificate	29	19.9
Diploma	87	59.6
Degree	29	19.9
Masters	1	0.7
**Duration of Practice (years)**		
≤ 5	32	21.9
6-10	24	16.4
11-15	27	18.5
16-20	21	14.4
21-25	23	15.8
26-30	18	12.3
≥ 30	1	0.7
**Marital status**		
Married	105	71.9
Separated	6	4.1
Single	22	15.1
Widowed	13	8.9
**Tribe**		
Indigenous Plateau tribe	124	84.9
Non-Indigenous Plateau tribe**	22	15.1
**Religion**		
Christian	114	78.1
Moslem	32	21.9
**Received training**		
Yes	20	13.7
No	126	86.3

* Afizere, Berom, Mwaghavul, Taroh, etc **Efik, Fulani, Hausa, Igbo, Yoruba

**Knowledge of COPD:** almost half (47.9%) of the health workers did not know the definition of COPD, almost 15% were able to state it correctly. The risk factor identified the most frequently was exposure to work dust or chemicals. The symptom that was most frequently identified was shortness of breath (93.8%). Less than 40% were able to identify cyanosis in the form of bluish/darkish discoloration of the lips as a symptom of COPD. Symptoms that are incorrect for COPD but were stated included diarrhoea (9.6%), fever (53.1%) and vomiting (30.8%). The test most frequently selected as a screening test for COPD was the chest X-ray. The peak flow meter was the second most selected followed by the stethoscope. Less than 20% of the respondents were able to correctly identify spirometry as the test used in the diagnosis of COPD with almost 14% stating that they did not know the diagnostic test for COPD. The mean score for the respondents´ knowledge of COPD was 13.9 ± 4.4, only 4.8% had good knowledge of COPD while 58.2% had fair knowledge. Details are shown in Table 2.

**Table 2:** knowledge of COPD and Asthma

COPD				Asthma		
Variable	Response	Frequency N = 146	%	Response	Frequency N = 146	%
**Definition**	Correct	20	13.7	Correct	50	34.3
	Incorrect	56	38.4	Incorrect	68	46.6
	Don't know	70	47.9	Don't know	28	19.2
**Risk factors (multiple responses)**	Work dust/chemicals	127	86.9	Air Pollution	132	90.4
	Tobacco Smoking	124	84.9	Smoke from Burning Wood/Charcoal	126	86.3
	Air Pollution	120	82.2	Family History	122	83.6
	Smoke from burning wood/charcoal	116	79.5	Tobacco Smoking	117	80.1
	Genetics	96	66.2	Genetics	100	68.5
	Low Birth Weight	33	22.6	Obesity	40	27.6
				Low Birth Weight	23	15.8
**Symptoms (multiple responses)**	Shortness of breath	137	93.8	Chest tightness	138	94.5
	Chest Tightness	136	93.2	Coughing	133	91.1
	Coughing	133	91.1	Shortness of Breath	145	99.3
	Wheezing	128	87.7	Wheezing	134	91.8
	Tiredness	112	76.7			
	Repeated chest infections	108	73.9			
	Sputum production	105	71.9			
	Bluish/darkish discolouration of the lips (cyanosis)	54	36.9			
**Symptoms/signs of an asthma attack**	–	–	–	Extreme Difficulty in Breathing	142	97.3
	–	–	–	Rapid Pulse	117	80.7
	–	–	–	Sweating	98	67.6
	–	–	–	Decreased Level of Alertness	87	59.6
	–	–	–	Severe Anxiety	82	56.2
	–	–	–	Bluish/Darkish Discolouration of the Lips	48	33.1
**Instruments used for screening (multiple responses)**	Chest x-ray	43	29.5	Stethoscope	46	31.5
	Peak flow meter	38	26	Chest X-ray	34	23.3
	Stethoscope	36	24.7	Peak flow meter	25	17.1
	Spirometer	11	7.5	Spirometer	14	9.6
	Standardized questionnaire	10	6.8	Blood test	6	4.1
	Blood test	9	6.2	Standardized questionnaire	6	4.1
**Diagnostic test**	Chest X-ray	61	41.8	Auscultation	46	31.5
	Auscultation	41	28.1	Chest X-ray	41	28.1
	Spirometry	24	16.4	Don't know	27	18.5
	Don’t know	20	13.7	Exercise test	3	2.1
				Spirometry	29	19.9
**Knowledge grade**	Good	7	4.8	Good	93	63.7
	Fair	85	58.2	Fair	21	14.4
	Poor	54	36.9	Poor	32	21.9
**Overall knowledge grade of COPD and asthma**	Good Fair Poor	6 100 40	4.1 68.5 27.4	-	-	-
**COPD mean knowledge score**	13.9 ± 4.4	p = 0.000		-	-	-
**Asthma mean knowledge score**	29.9 ± 6.8			-	-	-

Knowledge of Asthma Most of the respondents (47%) were not able to define asthma, while almost 20% stated that they didn´t know how to define it. The common risk factors for asthma were readily identified by the respondents (Table 2). Less common risk factors such as obesity (27.6%) and low birth weight (15.8%) were identified by a few of the respondents. Most of the respondents were able to identify the four common symptoms of asthma (i.e. chest tightness, coughing, shortness of breath and wheezing). Incorrect answers that were selected included fever (45.9%), vomiting (18.6%) and diarrhoea (5.5%). For asthmatic attacks, the symptom of extreme difficulty in breathing was readily identified by almost all the respondents (97.3%). The sign that was least identified was cyanosis (33.7%) which is visible as a bluish or darkish discolouration of the lips. For screening of asthma, the instrument stated by most of the respondents to be used is the stethoscope (31.5%). The use of the peak flow meter with a standardized questionnaire was stated by less than a quarter of the respondents. The diagnostic test stated most frequently was auscultation with a stethoscope (31.5%). The actual test for diagnosis, spirometry was identified by less than 20% of the study population. The mean knowledge score on asthma for the respondents was 16.1 ± 3.5. Most of the respondents (63.7%) had a good knowledge of asthma.

**Comparison of COPD and asthma knowledge:** most (58.2%) of the respondents had a fair knowledge of the CRDs under study. The difference in the mean knowledge scores of COPD and asthma were statistically significant (p = 0.000) with the mean scores of asthma being much higher than that of COPD. The mean total score of knowledge of asthma and COPD was 29.9 ± 6.8. There was a statistically significant relationship between the knowledge of health workers and age, sex and cadre as shown in Table 3.

**Table 3:** relationship between knowledge and socio-demographic characteristics of respondents

VARIABLE	Adequate knowledge N = 106 Freq (%)	Inadequate knowledge N = 40 Freq (%)	Total Freq (%)	p-value
**Age Group (years)**				0.02
20 - 30	24 (88.9)	3 (11.1)	27 (100.0)	
31 - 40	24 (58.5)	17 (41.5)	41 (100.0)	
41 - 50	35 (68.6)	16 (31.4)	51 (100.0)	
51 - 60	23 (85.2)	4 (14.8)	27 (100.0)	
**Sex**				0.02
Female	80 (68.4)	37 (31.6)	117 (100.0)	
Male	26 (89.7)	3 (10.3)	29 (100.0)	
**Cadre**				0.0015*
Nurse/Midwife	33 (97.1)	1 (2.9)	34 (100.0)	
CHO	8 (80.0)	2 (20.0)	10 (100.0)	
CHEW	34 (68.0)	16 (32.0)	50 (100.0)	
JCHEW	31 (59.6)	21 (14.4)	52 (100.0)	
**Highest Level of Education**				0.15*
Certificate	18 (62.1)	11 (37.9)	29 (100.0)	
Diploma	62 (58.5)	25 (28.7)	87 (100.0)	
Degree	25 (86.2)	4 (13.8)	29 (100.0)	
Masters	1 (100.0)	0 (0.0)	1 (100.0)	
**Duration of Practice (years)**				0.36*
≤ 5	25 (78.1)	7 (21.9)	32 (100.0)	
6-10	15 (62.5)	9 (37.5)	24 (100.0)	
11-15	16 (59.3)	11 (40.7)	27 (100.0)	
16-20	15 (71.4)	6 (28.6)	21 (100.0)	
21-25	19 (82.6)	4 (17.4)	23 (100.0)	
26-30	15 (83.3)	3 (16.7)	18 (100.0)	
≥ 31	1 (100.0)	0 (0.0)	1 (100.0)	
**Received Training**				0.06*
Yes	18 (90.0)	2 (10.0)	20 (100.0)	
No	88 (69.8)	38 (30.2)	126 (100.0)	

* Fisher exact

**Skill evaluation:** a majority of the respondents stated that there were no guidelines available for the screening, diagnosis or management of CRDs. Although almost 15% stated that guidelines were available, these were not sighted. Almost 90% had not screened nor referred for CRDs in the past one month. Less than 40% had provided health education on the diseases of interest, though about half had provided health education on the main risk factor shared by both diseases-tobacco smoking. None of the health workers were able to operate a peak flow meter. None were also able to state the recommended cut off value used for the screening of COPD and asthma and the method used to differentiate the two diseases by use of the peak flow meter (Table 4).

**Table 4:** skill evaluation of Health Workers

Variable	Response	Frequency (%)
**Availability of guidelines**	Yes	21 (14.4)
	No	125 (85.6)
**Number screened for CRD in past 1 month**	≥ 5	5 (3.4)
	< 5	14 (9.6)
	None	127 (86.9)
**Number referred for CRD in past 1 month**	≥ 5	5 (3.4)
	< 5	16 (10.9)
	None	125 (85.6)
**Provided health education on:**		
Asthma	Yes	51 (34.9)
COPD	Yes	33 (22.6)
Smoking	Yes	74 (50.7)
**Ability to operate peak flow meter**	Yes	0 (0.0)
	No	100 (100.0)
**Recommended value for diagnosis of COPD with use of peak flow meter**	Correct	0 (0.0)
	Incorrect	100 (100.0)
**Ability to differentiate asthma from COPD using a peak flow meter**	Yes	0 (0.0)
	No	100 (0.0)

## Discussion

The study determined that 58.2% of respondents had a fair knowledge of COPD and asthma with knowledge of asthma being significantly better than that of COPD. Factors associated with knowledge were younger age, male sex and more senior-level cadre. Most respondents also lacked the necessary skills to screen for COPD and asthma.

Knowledge of COPD: the importance of accurate knowledge for health workers cannot be overemphasized especially at the primary level where they are expected to provide health education on common ailments within communities [31]. Few were able to correctly provide a definition for COPD. A study among nurses had a similar result with majority of the nurses providing incorrect definitions of COPD [32]. Although many of the respondents correctly identified the risk factors for COPD, they also stated many risk factors that are not related to COPD indicating that there may be misconceptions among them regarding the disease. Almost 15% of respondents did not know smoking is a risk factor for COPD. Smoking is the main risk factor for the occurrence of COPD and it has been shown to be prevalent within the study area [33,34]. Hence it would negatively affect the control of this disease if health workers are not aware of the association between this disease and tobacco smoking and even more so as it is also a risk factor for asthma. Similar to their knowledge of the risk factors, quite a number still stated incorrectly certain symptoms that are not related to COPD. The symptom that indicates cyanosis was also not readily stated as a symptom. This may be because the bluish or darkish discoloration of the lips is not easily recognizable in dark skinned people and may require an experienced eye to detect it. There was also a gap in knowledge regarding screening and diagnostic tools for COPD. A similar study conducted among a variety of primary care workers also found their knowledge to be “intermediate&lrquo; in these same areas of risk factors, symptoms, and diagnostic tools [32]. The level of knowledge in this study may be due to lack of training to refresh and update their knowledge as it was noted that the training mentioned here was the basic knowledge acquired in their schools of training. In-service training would have helped to address those gaps in their knowledge. Although COPD is a disease that is managed mostly by respiratory medicine experts, due to its economic burden, its symptoms and criteria should also be known by primary health care providers who are frontline health providers especially in rural areas. This will lead to prevention such as smoking cessation and early diagnosis to avoid complications, thus halting patients proceeding to advanced stages of the disease that mainly requires symptomatic and expensive approaches [35].

**Knowledge of asthma:** most of the health workers had a fair knowledge of asthma. However many were not able to provide a sufficient definition for asthma as would be expected from health care providers. This is similar to a finding that was conducted among nurses where most of them could not define asthma [36]. Common risk factors for asthma were easily identified by most respondents. However, few were able to identify less known risk factors which included low birth weight and obesity. Obesity is linked to so many non-communicable diseases and it would be of benefit to patients to realize that asthma especially manifesting in adulthood can be as a result of obesity [37]. The most frequent answer provided as a risk factor was air pollution. This may be because in this environment there is an increase in presentation of asthma during the harmattan season which is characterized by a high content of dust and pollen in the atmosphere. Most of the respondents were able to readily identify the symptoms that asthma patients present with which is not surprising as the symptoms of asthma are easily recognizable. However, symptoms that are not associated with asthma were stated. These misconceptions may cause misdiagnosis or even lead health workers to provide inaccurate information to patients and community members. So also, the symptoms of an asthmatic attack were readily stated by the respondents with the exception of the symptom that indicates cyanosis as was seen in the answers provided for COPD. Less than 20% knew of the use of the peak flow meter and a standardized questionnaire for screening which is the recommended test at primary level and the use of spirometry for diagnosis which is the gold standard. Since these tools are the requirement for identifying those with the disease and referring adequately, it implies that the health workers are not aware of what is expected of them to identify such patients when they present and thereby provide adequate management and referral.

**Factors affecting knowledge of COPD and Asthma:** in this study, knowledge was found to be associated with age, sex and cadre. These findings are similar to those in another study where occupation, age and gender were found to influence knowledge of asthma [29] and also with another study on the knowledge of COPD that found age and years of experience to be associated with better knowledge [35]. In this study, those in the age group of 20-30 years had more knowledge than the other groups. This may be because this is the group most recently out of school and was relying on their residual knowledge. Those in the age group 51-60 also had a high proportion of adequate knowledge. This may be as a result of the years of experience as they would have been exposed to more patients with these diseases. Males were found to have better knowledge than the females. This contrasts with the study stated above where the females were found to have better knowledge of asthma [29]. Although this was not further explored in the results, it may be that the males collectively have had longer years of experience, or have higher levels of education which are factors that have been known to influence knowledge. Among the cadres, the proportion of those with adequate knowledge increased with the level of the cadre with the nurses/midwives ultimately having more knowledge than the other cadres. Training is usually found to improve knowledge but that was not the case in this study. This may be because all those that reported that they were trained were referring to the basic training that was given to them during school. No one had had additional training afterwards.

In this study that the knowledge of asthma was significantly higher than that of COPD. This infers that health workers are not very familiar with COPD. With the presentations of asthma and COPD being so similar, it is very possible that cases of COPD are being missed due to the lack of knowledge of health workers.

**Skills of health workers for screening:** the skill of the health workers was assessed for the ability to screen with the peak flow meter. Although about 20% stated that guidelines were available, this was not confirmed either by the facility heads or by the sighting of any copy of the guidelines. This is not surprising because even though Nigeria has a policy for NCDs, it is only available online in draft form, but not disseminated to states for adoption and ratification and neither has implementation commenced [38]. In respect to NCDs, no mention was made of CRDs and the only inclusion in the policy that will be useful is the guideline on tobacco smoking. None of the health workers knew how to use a peak flow meter and in most cases stated that they did not know what it was and additionally did not know the recommended values and how to go about differentiating between asthma and COPD using the same instrument. Consequently, very few had either screened for asthma or COPD or referred to a higher level. This may also mean that the health workers used symptomatic assessments to screen and subsequently refer. The use of the peak flow meter for screening, follow up and even home self-monitoring of patients with CRDs is a well-established practice in a number of countries [39,40]. One of the gaps that was identified by a multi-country study conducted in the east Mediterranean region on the integration of NCDs into primary health care was the lack of skill by the health workers to tackle these diseases [41].

**Limitations:** the study was conducted in one local government of a State of Nigeria and among a particular level of health care workers. The findings might therefore not be generalizable to all populations of health workers in the State and country.

## Conclusion

The results from this study have shown that the knowledge of COPD and asthma among primary health care workers in Jos North is mostly fair. However, there is a significant difference in the levels of knowledge as the average score were higher for asthma than for COPD. Despite this fair knowledge, there were gaps and misconceptions in their knowledge regarding the risk factor, symptoms, screening and diagnostic tests for both COPD and asthma. The health workers had no skill for the screening of COPD and asthma as most were not able to identify the screening tools and none could demonstrate the use of the peak flow meter. They also were mostly not able to identify the diagnostic test for both diseases. The study, therefore, concludes that there is a lack of adequate preparedness of primary health care workers for the screening and diagnosis of asthma and COPD.

### What is known about this topic

- That COPD and asthma are diseases with high morbidity and mortality;
- COPD and asthma are very similar in presentation and may be difficult to differentiate without use of recommended tools;
- There are tools available developed by WHO for resource-constrained countries to use to screen patients at primary health care level so that early management can be implemented.

### What this study adds

- An assessment of the readiness of PHC workers to provide useful information to communities and to readily pick out those with early onset of COPD and asthma An identification of the specific gaps in knowledge and skill required by the PHC workers in the area of study that can assist government authorities to better prepare their staff for the prevention of COPD and asthma in this community.
